# Genomic identification of direct seeding and evolutionary lineages by combining heterogeneous genomic resources

**DOI:** 10.1186/s12915-025-02476-5

**Published:** 2025-12-12

**Authors:** Jasmine Noëlle Tschan, Oliver Reutimann, Simone Fior, Amandine Cornille, Anamaria Roman, Tudor-Mihai Ursu, Alex Widmer, Martin C. Fischer

**Affiliations:** 1 Institute of Integrative Biology, ETH Zurich, Zurich, Switzerland; 2 Division of Science, New York University Abu Dhabi, Saadiyat Island, Abu Dhabi, United Arab Emirates; 3 Institute of Biological Research Cluj, National Institute of Research and Development for Biological Sciences, Cluj-Napoca, Romania

**Keywords:** Conservation genetics, *Dianthus carthusianorum*, ddRAD, Direct seeding, Evolutionary significant unit (ESU), Seed provenance, Whole genome re-sequencing (WGS)

## Abstract

**Background:**

Human-induced habitat changes threaten biodiversity, prompting large-scale restoration initiatives. Revegetation through direct seeding is common in agricultural and infrastructure construction projects, yet the provenance of seed material and its genetic impacts on natural populations remain underexplored. Introducing foreign ecotypes can lead to unintended consequences, as they may be adapted to different environmental conditions or represent distinct evolutionary lineages. In Switzerland, direct seeding is widely used to promote dry meadows, often using seeds of the Carthusian pink (*Dianthus carthusianorum*).

**Results:**

To assess the extent and genetic effects of direct seeding and infer seed provenances, we combined genomic data from 446 samples collected in independent, smaller-scale studies. We assembled a chromosome-level reference genome to map reads and developed a panel of 48,299 representative single nucleotide polymorphisms (SNPs). We identified six evolutionary significant units (ESUs) within the European distribution range of *D. carthusianorum*. As biodiversity promotion efforts are often coordinated nationally, we focused on populations in Switzerland, where we found five ESUs: four occur naturally, and one was introduced from Eastern Europe. Our combined genomic data revealed that 15 of 31 randomly sampled populations across Switzerland (48.4%) originated from direct seeding. Allochthonous seed material was detected in eight populations (25.8%), with six of these showing admixture involving two to three ESUs.

**Conclusions:**

Our results demonstrate the effectiveness of genomic approaches for identifying direct seeding and clarifying seed provenance, thereby supporting decision-making in national revegetation projects and emphasising the importance of using autochthonous seed sources.

**Supplementary Information:**

The online version contains supplementary material available at 10.1186/s12915-025-02476-5.

## Background

Human activities during the Anthropocene have significantly impacted nature, primarily through land-use changes, resource exploitation, pollution, the introduction of invasive alien species, and climate change. The impact of such activities is increasing the risk of biodiversity loss at all three levels: the diversity of ecosystems, species diversity, and genetic diversity within species. Since the Industrial Revolution, an estimated 6–30% loss of species’ genetic diversity has occurred [1, 2]. Currently, the existence of around one million species is threatened [3], and habitats for terrestrial organisms are deemed insufficient for the long-term survival of over 500,000 species [3]. Hence, biodiversity promotion and restoration are needed. National and international authorities have recognised the urgency of acting against biodiversity loss and agreed at the 15th Conference of the Parties (COP15) of the Convention on Biological Diversity (CBD; [4]) on a Global Biodiversity Framework (GBF; [5]) and to actively promote biodiversity. In particular, to reverse biodiversity loss caused by land-use change, national and international authorities are called upon to implement conservation measures and achieve biodiversity targets by protecting and connecting habitats, controlling invasive species, and translocating or reintroducing species for habitat restoration [6, 7].

The United Nations’ call for a ‘Decade on Ecosystem Restoration’ [8] and the Sustainable Development Goals (SDGs), in particular Goal 15, which focuses on protecting and restoring ecosystems worldwide [9], have extensively promoted revegetation measures such as direct seeding or planting seedlings. These are effective ways to promote target species, increase plant coverage, restore degraded habitats, preserve soil, store carbon, increase biodiversity, and limit the spread of invasive species [10]. Re-established vegetation provides habitat and food for organisms at higher trophic levels [11] and helps to develop healthy ecosystems, which are essential to prevent biodiversity collapse [12]. However, the provenance of the revegetation material is often unknown, and the use of allochthonous (non-local) provenances can impede the success of revegetation projects [10, 13].

Past glacial cycles in the northern hemisphere have forced species into different refugia, greatly influencing species distributions (e.g. [14–16]). During the Last Glacial Maximum and previous glacial cycles, many European species survived in southern European refugia and expanded to the North after the retreat of the glaciers [15]. Repeated cycles of range expansion and contraction during glacial periods have shaped patterns of genetic divergence and population structure, likely through allopatric divergence, contributing to the formation of distinct evolutionary lineages. In addition, post-glacial recolonisation can lead to lower genetic diversity in populations that are further away from the historic refugium, due to recurrent bottlenecks at the front of the expansion (e.g. [17, 18]). Patterns of isolation and divergence can leave detectable signatures in population structure, which are commonly used in conservation genetics to delineate evolutionary significant units (ESUs; [19, 20]). Such ESUs are conservation-relevant as they can harbour adaptive genetic diversity, contributing to a species’ evolutionary potential [21]. As a result, seeds or seedlings from different provenances can differ in their reproductive capacity, genetic diversity, and adaptive traits [22, 23]. These genetic factors can negatively influence the population dynamics of newly seeded populations if the populations are not adapted to their new habitat. Introducing individuals from genetically distinct ESUs that are not locally adapted can pose risks such as maladaptation to local conditions or inbreeding. This can put the development of healthy populations and ecosystems at risk. Gene flow from allochthonous ESUs into local populations can further lead to outbreeding depression, which manifests as reduced expression of fitness-related traits, including lower seedling emergence, decreased flower production, and lower biomass [13, 22, 24–28]. Conversely, introducing new genotypes that hybridise with the local populations can mask genetic load and potentially increase fitness. This can be used as a tool for the genetic rescue of populations with low genetic diversity [29]. Therefore, hybridisation and introgression of divergent ESUs can potentially have both positive and negative consequences, which are often species-specific and difficult to predict, thus requiring further monitoring and study. Although best practices for direct seeding are being developed, little is known about the extent of its application over recent decades or about the genetic consequences for local wild populations. Molecular genetic tools could offer powerful means to retrospectively detect past direct seeding events, identify seed sources, and assess patterns of introgression between natural and introduced populations.

Most conservation efforts are coordinated at local to national scales because of legal and financial constraints, and therefore only cover part of the distribution range of most species. However, this narrow geographic focus could limit our understanding of the species’ overall genetic structure and diversity, which can be highly relevant for effective conservation planning [30, 31]. Numerous rapidly growing data repositories, such as the International Nucleotide Sequence Database Collaboration (INSCD; [32]), provide open access to genomic data from other studies. Such resources allow expanding the geographic range of sampling and cost-effective acquisition of genetic data to assess genetic structure across a large part of the species range and potentially distinguish ESUs. Given the high-throughput sequencing (HTS) revolution [33], the most commonly used data nowadays are HTS-based single nucleotide polymorphisms (SNPs) from different sequencing methods [34], such as restriction-site associated DNA sequencing (RAD-Seq; [35]) or whole genome re-sequencing (WGS; [36]). Lower sequencing costs have made various sequencing methods more accessible, increasing the availability of high-quality reference genomes and enabling more accurate mapping and improved genotyping quality [37]. Leveraging the value of different existing sequencing datasets requires combining them without introducing technical biases that could affect downstream analyses. Such biases may arise from differences in primary base calling of the sequencers, such as the shift from 4-channel to 2-channel chemistry in Illumina [38–40], or variations in sequencing length that could result in batch effects within the data of the same study, affecting downstream genotyping [41]. Furthermore, bioinformatic processing and filtering can lead to biases [42]. Hence, suitable SNP calling and filtering strategies are necessary to combine genomic resources effectively and accurately, thereby expanding the geographic coverage of regional or national conservation genetics projects.

Direct seeding is widely applied in Switzerland to promote dry meadows, which are habitats of national importance [43]. Dry meadows are among the country’s biodiversity hotspots, with up to 100 species per 100 m^2^, harbouring 13.1% of Switzerland’s plant species diversity [44]. In Switzerland, dry meadows have suffered a severe decline of around 95% over the last century due to the intensification of agriculture, expansion of settlements, reforestation, and succession following the abandonment of traditional land management [45]. To counteract this rapid decline, Switzerland has widely applied direct seeding of plant species typical of dry meadows over the past two decades to promote biodiversity, prevent soil erosion, restore habitat quality, control invasive species, and enhance landscape attractiveness [46]. One emblematic species of dry meadows is the commonly diploid Carthusian pink (*Dianthus carthusianorum* L.), mainly distributed within the mountainous regions of central Europe, ranging from Germany to Italy in latitude and from Ukraine to Spain in longitude [47–49]. In Switzerland, this butterfly-pollinated, gynodioecious species, in which hermaphrodites are self-compatible but predominantly outcrossing, is frequently used in seed mixtures for dry meadows, agricultural ruderal sites, and urban or roof greening [50–52]. Seed mixtures can be obtained from large-scale seed suppliers that propagate seeds with origins aligned to the country’s biogeographical regions. Due to its frequent use in restoration projects, *D. carthusianorum* was one of five species selected in a pilot study for a monitoring of genetic diversity in Switzerland [53]. A total of 310 individuals from 31 randomly sampled populations across Switzerland were whole-genome re-sequenced, providing an ideal dataset to study the extent and genetic effects of direct seeding for biodiversity promotion.

To assess whether *D. carthusianorum* encompasses different ESUs, which may be conservation-relevant, and whether allochthonous seed material was used in direct seeding efforts, it is essential to examine the species’ distribution range beyond national borders. To achieve this, we combined samples from different studies and sequencing types [53, 54], and added new samples to fill gaps in the distribution range. We focused on the following research questions: (i) How can heterogeneous genomic resources from different studies be integrated to expand sampling across species’ distribution ranges to identify evolutionary lineages and ESUs? (ii) Which ESUs of *D. carthusianorum* occur naturally in Switzerland, and which have been introduced? (iii) How can we use genomic data to identify direct seeding and seed origin? (iv) What is the prevalence and abundance of direct seeding in Switzerland? (v) How many years ago were the direct-seeded populations, identified through genomic data, originally established? (vi) Has hybridisation and introgression occurred between natural and direct-seeded ESUs, and how often? (vii) How do levels of genetic diversity differ among ESUs, and is genetic diversity lost during seed production for restoration purposes?

## Results

### *Dianthus carthusianorum* reference genome

The genome size of *D. carthusianorum* was estimated at approximately 540 Mb based on *k*-mer analysis (Additional File 1: Figure S1). The newly assembled reference genome haplotype 1 (ethDiaCart_GR_1.1) has a length of 498,671,606 bp and consists of 62 scaffolds with an N50 of 33,218,876 bases (Additional File 1: Supporting Methods [55–62], Supporting Results, Table S1 and Additional File 2). The sequence length N90 was 30,263,282 bases, and the L90 value corresponded to 14 contigs. All 15 haploid chromosomes of *D. carthusianorum* are represented in the assembly based on Omni-C scaffolding (Additional File 1: Figure S2). The reference genome contained 40,047 annotated genes and coding regions of a total length of 70,272,860 bases. Of the 2326 BUSCO genes searched (eukaryota_odb10 dataset), 93.5% were complete, of which 88.2% were single-copy and 5.3% were duplicated.

### In silico SNP panel, SNP sets, and genotyping error

The newly developed SNP panel based on 136 *D. carthusianorum* ddRAD-SE and -PE samples included 48,299 SNPs. The *EvoLin SNPs*, designed to identify evolutionary lineages and assess genetic divergence within *D. carthusianorum*, were called and filtered using this SNP panel and 198 individuals, resulting in 42,466 SNPs. After linkage disequilibrium (LD) thinning to one SNP per 5 kb, 6307 unlinked SNPs were retained. The *Seeding detection SNP* set, tailored explicitly for detecting direct seeding and admixture events within Swiss populations, based on the SNP panel and 310 Swiss samples, resulted in 33,725 SNPs after filtering (6106 after LD-thinning). The *Phylogenetic SNP*s, intended to explore broader evolutionary relationships, including five related *Dianthus* species, were based on a de novo variant calling that contained 10,209 SNPs.

### Evaluating the combination of genomic resources

The overall genotyping error rates varied slightly between the different sequencing methods but were generally low, with 0.38% for ddRAD technical replicates, 0.29% for WGS technical replicates, and 1.08% for SNPs called from ddRAD and WGS data (Table 1). The mean observed heterozygosity (*H*_O_) estimated from ddRAD data differed from that estimated from WGS data by 3.22% (Additional File 1: Figure S3).

**Table 1:** Genotyping error rates (in %) estimated from technical replicates: eight ddRAD replicates, five WGS replicates, and 31 samples analysed with both WGS and ddRAD

Error rate type	ddRAD [%]	WGS [%]	ddRAD vs WGS [%]
Genotyping	0.38	0.29	1.08
Homozygous positions	0.04	0.12	0.37
Heterozygous positions	4.02	1.67	5.77

### Identification of evolutionary lineages and ESUs

**Fig. 1:**
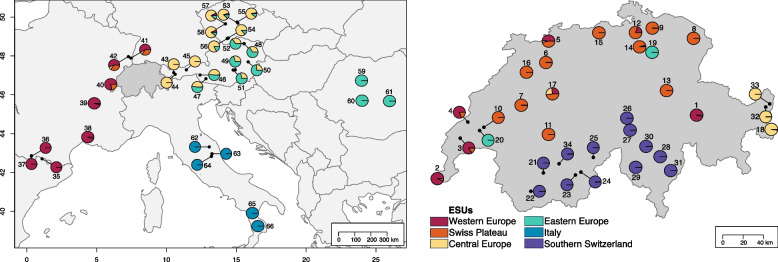
Geographic distribution of 66 European *Dianthus carthusianorum* populations. Pie charts illustrate the genetic structure of each population based on admixture analysis (*K* = 6). Each colour represents one of six inferred evolutionary significant units (ESUs), with proportions indicating the relative genetic contribution of each ESU. Population numbers correspond to Additional File 3

We investigated population genetic structure and admixture within *D. carthusianorum* using the *EvoLin SNPs*. The elbow shape of the ADMIXTURE cross-validation error plot (Additional File 1: Figure S4) indicated that our data best support six distinct evolutionary lineages (*K* = 6). The ancestry proportions inferred by ADMIXTURE were consistent in 86 of 100 runs across Europe (Fig. 1; *K* = 2–10 in Additional File 1: Figure S5). Throughout this manuscript, we consistently refer to these lineages as ESUs, a classification decision supported by genetic clustering and subsequent analyses of strong genomic divergence and historical genetic isolation inferred from the phylogenetic relationships presented later in this study. The ESUs are hereafter named according to their geographic locations: Southern Switzerland, Swiss Plateau, Italy, Central Europe, Eastern Europe, and Western Europe.

**Fig. 2:**
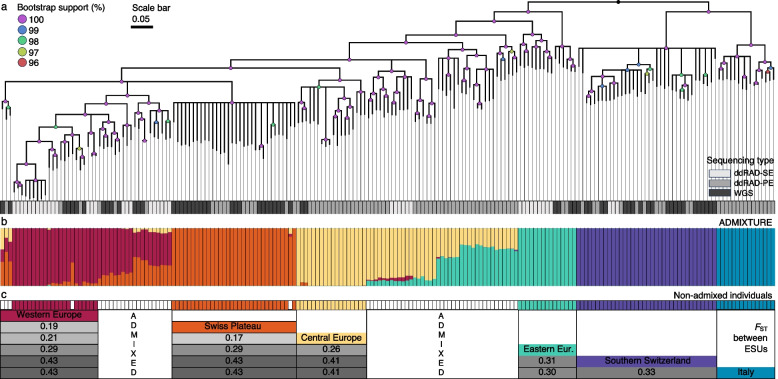
**a** Phylogenetic RAxML tree of 198 *Dianthus carthusianorum* individuals from across the European distribution range. The tree was inferred with ascertainment bias correction [63] to account for SNP-only data; branch lengths represent relative divergence among lineages rather than absolute substitution rates. The genotyping involved three different types of genomic sequencing, indicated by various shades of grey. **b** Results of ADMIXTURE analysis (*K* = 6) and bar plot of estimated individual ancestry coefficients coloured according to the six evolutionary significant units (ESUs): Western Europe (red), Swiss Plateau (orange), Central Europe (yellow), Eastern Europe (turquoise), Italy (blue), Southern Switzerland (violet). (c) Pairwise *F*_ST_-Matrix between the non-admixed evolutionary significant units (ESUs) are indicated by a colour gradient from low (light grey) to high (dark grey).

The six ESUs inferred by ADMIXTURE were further supported by the topology of the phylogenetic tree based on 38,141 SNPs (Fig. 2a), with individual-level admixture patterns largely matching the phylogenetic clustering (Fig. 2b). Nearly identical patterns were also found in the LD-thinned *EvoLin SNPs* (one SNP per 5 kb; 6307 SNPs), for which the individual admixture results are provided in Additional File 1: Figure S4. The relative genetic divergence, as measured by pairwise *F*_ST_, between ESUs (after excluding individuals with > 10% admixture), was found to be as high as 0.43 between Italy and Western Europe and between Southern Switzerland and the Swiss Plateau. The lowest divergence was observed between the ESUs of Western and Central Europe, with an *F*_ST_ value of 0.17 (Fig. 2c).

**Fig. 3:**
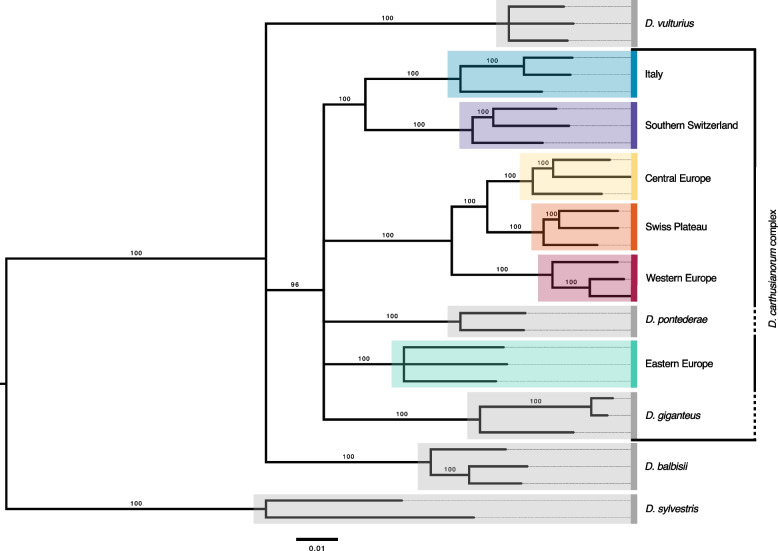
Phylogenetic RAxML tree illustrating the relationships within the *Dianthus carthusianorum* complex. The six evolutionary significant units (ESUs) of *D. carthusianorum* are depicted in colour, while five closely related *Dianthus* species are shown in grey. Only nodes with bootstrap support > 10% are shown; nodes with lower support were collapsed. The scale bar indicates nucleotide substitutions per site. Because the phylogeny is based on SNP-only data, branch lengths were estimated under an ascertainment bias correction in RAxML [63] and should be interpreted as relative divergence among lineages, not as absolute substitution rates. Geographical origins of outgroup samples are shown in Additional File 1: Figure S6, and coordinates are provided in Additional File 3

The phylogenetic RAxML tree (Fig. 3), based on 6325 SNPs and including five closely related *Dianthus* species, as well as non-admixed individuals from each of the *six D. carthusianorum* ESUs, revealed that *D. giganteus* and *D. pontederae* form a monophyletic group with the six ESUs of *D. carthusianorum*. Despite being phenotypically and ecologically distinct, these two taxonomically well-described species cluster within the *D. carthusianorum* complex. *Dianthus vulturius* and *D. balbisii* are phylogenetically distinct.

In summary, the six evolutionary lineages, distinguished by strong genome-wide divergence, were defined as ESUs. Their delineation is consistently supported by genetic differentiation and phylogenetic analyses (Figs. 1, 2, and 3).

### Identification of direct seeding in Switzerland

Of the six ESUs identified across Europe, five were present in Switzerland, of which four occur naturally (Fig. 1). The fifth, a non-native ESU from Eastern Europe, was detected in two populations (19 and 20), which were classified as allochthonous direct seeding. In addition, one population (01) in eastern Switzerland was assigned to the Western European ESU. This ESU is native to Switzerland but does not naturally occur in the eastern part of the country. Because this population was more than 100 km beyond the ESU’s range border, it was classified as being of allochthonous origin. Overall, three populations sampled in Switzerland were therefore classified as allochthonous.

**Fig. 4:**
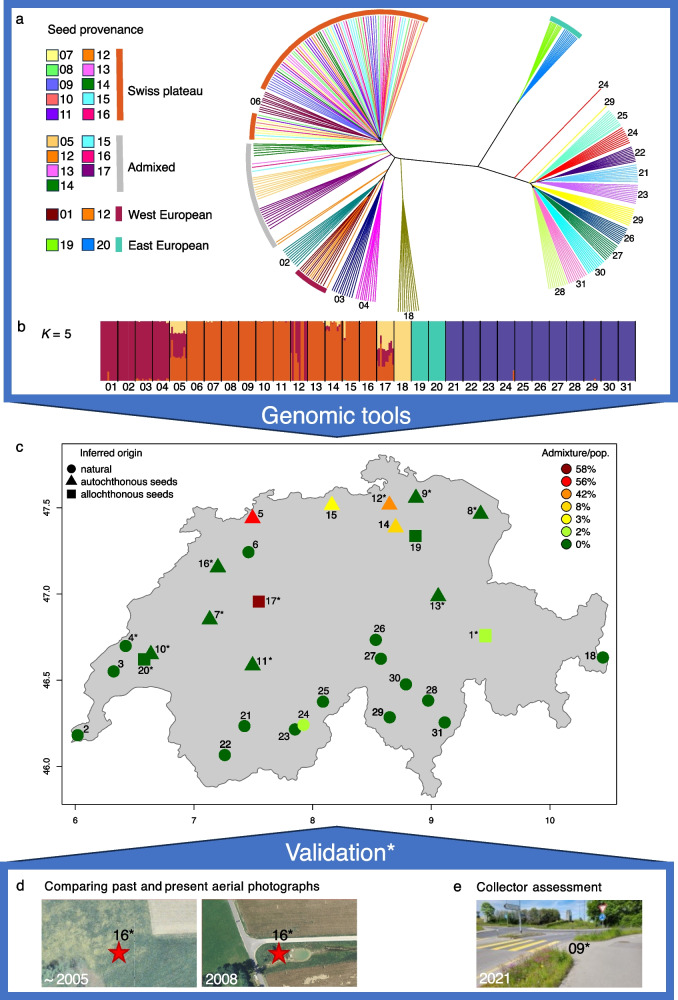
Stepwise procedure to infer and validate direct seeding in Switzerland. **a** Unrooted phylogenetic tree based on an individual pairwise dissimilarity matrix. Colours and numbers represent *Dianthus carthusianorum* populations (see Table 2 and Additional File 3). Coloured bars on terminal branches indicate seeded provenances; natural populations are unmarked. Because some populations are admixed or contain non-admixed individuals from different ESUs, certain population numbers appear more than once. **b** Admixture analysis based on a Swiss-specific genetic clustering (*K* = 5), identifying five evolutionary significant units (ESUs) within the Swiss dataset, each represented by a distinct colour. **c** Map of Swiss *D. carthusianorum* populations, showing sites inferred as affected by direct seeding with autochthonous ESUs (triangles), allochthonous ESUs (squares), and natural populations not inferred as seeded (circles). Admixture per population is indicated by a green-to-red gradient (0–58%). **d**, **e** Asterisks (*) mark populations validated as seeded through **d** aerial photographs (historical habitat alteration) or **e** on-site assessment by seed collectors (see Table 2)

Using an individual pairwise dissimilarity matrix visualised as a phylogenetic tree (Fig. 4a), we identified four genetically distinct seed sources in Switzerland: (i) homogeneous seed sources from the Swiss Plateau, (ii) Western Europe, (iii) Eastern Europe, and (iv) admixed sources composed of seeds from 2 to 3 ESUs. In this tree, individuals from homogeneous seed sources formed star-shaped clusters, grouping across populations rather than within them. This clustering pattern alone allowed us to classify 11 populations as direct-seeded from homogeneous sources, independent of other methods.

**Table 2:** Summary of non-genomic and genomic evidence for direct seeding of *Dianthus carthusianorum* in Switzerland. ‘Pop no.’ corresponds to population numbers in Figs. 1 and 4, and Additional File 3. Under *Non-genomic evidence*, ‘Collector assessment of seeding’ (X) indicates populations for which collectors indicated that they probably originated from direct seeding, and ‘Habitat modification (years)’ refers to the time periods during which a change in land use occurred, often as a result of infrastructure or restoration projects (Additional File 1: Figure S8). Under *Genomic inference of direct seeding*, ‘Seed provenance’ indicates the ESU identified as seed source; ‘Allochthonous’ (X) indicates populations established from allochthonous seed sources; ‘Admixed’ (X) indicates the presence of at least one individual with > 10% admixture from another ESU; and ‘Direct seeding’ (Yes/No) summarises the final classification for each population, which is based solely on genomic results; non-genomic evidence was used for comparison and cross-validation. Only populations inferred as seeded by at least one line of evidence are shown

	***Non-genomic evidence***		***Genomic inference of direct seeding***			
*Pop no*	*Collector assessment of seeding*	*Habitat modification (years)*	*Seed provenance*	*Allochthonous*	*Admixed*	*Direct seeding*
*1*		2013^†^	Western Europe	X	X	Yes
*4*	X					No
*5*			Admixed (Swiss Plateau + Western Europe + Central Europe)	X	X	Yes
*7*	X	1998–2004	Swiss Plateau			Yes
*8*	X		Swiss Plateau			Yes
*9*	X	2002–2005	Swiss Plateau			Yes
*10*	X		Swiss Plateau			Yes
*11*	X	2010–2013	Swiss Plateau			Yes
*12*	X	2016–2019	Admixed (Swiss Plateau + Western Europe), Swiss Plateau, Western Europe	X	X	Yes
*13*	X	2019–2021	Swiss Plateau			Yes
*14*			Admixed (Swiss Plateau + Central Europe), Swiss Plateau	X	X	Yes
*15*			Swiss Plateau, Central Europe	X	X	Yes
*16*	X	2005–2008	Swiss Plateau			Yes
*17*	X	1993–1998	Admixed (Central Europe + Swiss Plateau + Western Europe)	X	X	Yes
*19*			Eastern Europe	X		Yes
*20*	X	2017–2020	Eastern Europe	X		Yes
*24*					X	No

^†^For these sites, the year during which the populations were established was known precisely

Individual ADMIXTURE analysis of Swiss populations revealed recent admixture in seven cases, each containing at least one individual with more than 10% ancestry from another ESU (Fig. 4b). Five of these populations (05, 12, 14, 15, 17) involved at least one allochthonous ESU and were therefore classified as direct-seeded, consistent with introduction through human-mediated seeding rather than natural dispersal. In contrast, one population (24) showed admixture with an autochthonous ESU, indicating that this likely resulted from natural gene flow. The highest admixture proportions were found in populations 05 and 17 (Fig. 4c), with 56% and 58% admixture across three ESUs (Swiss Plateau, Central Europe, and Western Europe). In total, five admixed populations were attributed to direct seeding and one to natural processes.

Analyses based on the LD-thinned *Seeding Detection SNPs* (1 SNP per 5 kb; 6106 SNPs) recovered identical cluster assignments and frequencies, with a slight increase in low-level ancestry signals (< 10%). This is supported by consistent admixture patterns and a high correlation of Q-values between datasets (Additional File 1: Figure S7).

In summary, 15 of the 31 randomly sampled *D. carthusianorum* populations (48.4%) in Switzerland were identified as direct-seeded (Fig. 4c). Overall, three populations (9.7%) were derived from allochthonous seeds (01, 19, 20), five populations (16.1%) were direct-seeded with admixed seeds, and seven populations (22.6%) were direct-seeded with autochthonous homogeneous seed sources. The remaining 16 populations (51.6%) showed no evidence of direct seeding.

To compare and cross-validate the genomic inference of direct seeding, we incorporated two non-genomic approaches: collector assessments from 2021 field sampling and historical aerial photographs (Fig. 4d,e). Collector assessments presumed direct seeding in 35.5% (11 populations), including one case (04) that was not confirmed by either genomic inference or aerial photographs (Table 2). In contrast, 12.9% of all populations (05, 14, 15, 19) could only be detected as seeded using genomic tools, all of which were located in agricultural landscapes. Aerial photographs revealed human-mediated habitat modifications in nine populations, spanning 0–28 years before the 2021 sampling (mean 11.5 years). Six cases followed infrastructure construction or modification, and three followed river or landscape restoration (Additional File 1: Figure S8). All populations identified from aerial photographs as direct-seeded were also classified as such by our genomic approach.

### Genetic diversity and inbreeding of the natural and seeded ESUs in Switzerland

**Fig. 5:**
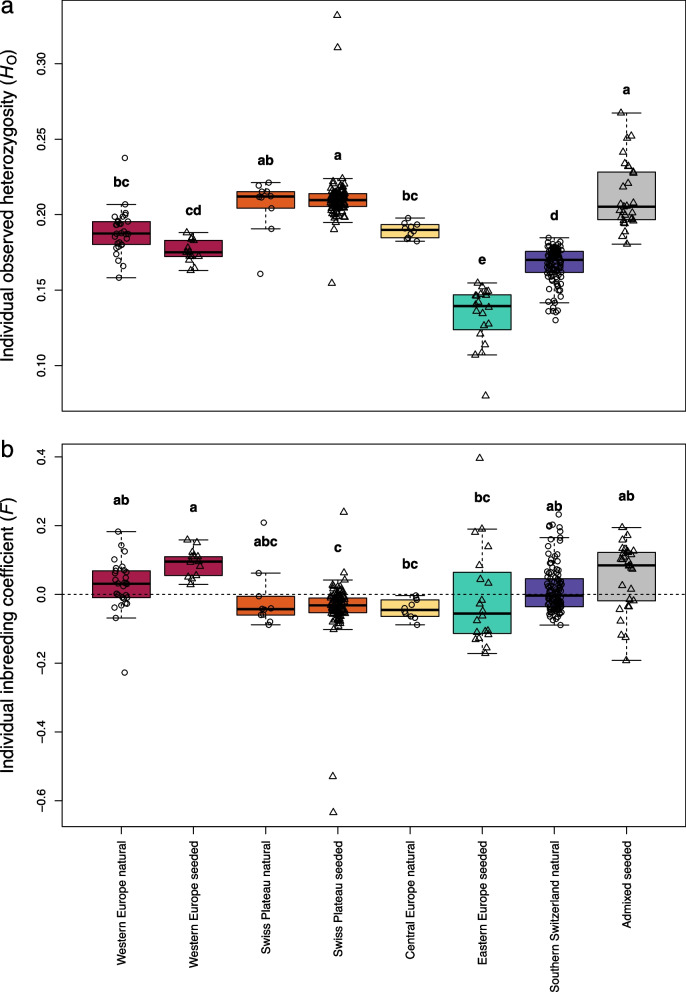
Genetic diversity and inbreeding in Swiss *Dianthus carthusianorum* evolutionary significant units (ESUs) and admixed individuals. **a** Individual site-specific observed heterozygosity (*H*_o_) and **b** individual inbreeding coefficient (*F*) for each ESU, shown separately for natural and seeded populations, and for the admixed group. Inbreeding coefficients were estimated within groups to minimise confounding by population structure. Colours indicate the different ESUs. Circles represent natural populations and triangles represent seeded populations. Letters show Tukey HSD groupings after ANOVA (*H*_O_: *p* < 2.0 × 10⁻^1^⁶;* F*: *p* = 1.5 × 10⁻⁸); groups sharing a letter do not differ significantly, whereas groups without a common letter do

To evaluate the genetic diversity of the ESUs and their conservation implications in Switzerland, we assessed individual observed heterozygosity (*H*_o_; Fig. 5a) and individual inbreeding coefficients (*F*; Fig. 5b). *H*_o_ differed significantly between ESUs, but not between natural and seeded individuals within each ESU (*p* < 2.0 × 10⁻^1^⁶, ANOVA; post hoc Tukey HSD corrected for multiple testing; Additional File 1: Figure S9a). The lowest *H*_o_, with a mean of 0.13 and 0.17, was found in the seeded populations from Eastern Europe and the natural populations from Southern Switzerland, respectively. The highest *H*_o_ value, with 0.21, shows the natural and seeded populations of the Swiss Plateau ESU. Mean individual inbreeding coefficients (*F*), calculated within ESUs, varied considerably among the ESUs (range − 0.043 to 0.093; Fig. 5b). There was a significant difference in *F* among ESUs, but not between natural and seeded populations within ESUs (*p* = 1.5 × 10⁻⁸, ANOVA; post hoc Tukey HSD corrected for multiple testing; Additional File 1: Figure S9b). The Central European ESU showed the lowest mean *F* with − 0.043. The Eastern Europe ESU had a mean *F* ≈ 0 but contained the individuals with the highest individual inbreeding with *F* up to 0.4. Individual *F* was also relatively high in Southern Switzerland and the Swiss Plateau, with several individuals having *F* > 0.2.

## Discussion

We integrated genotypic data from diverse HTS approaches across independent studies to identify high-quality SNP sets to assess genetic variation across a representative portion of the geographic distribution range of *D. carthusianorum*. This approach allows sampling to be extended beyond the regional or national scale typical of many conservation efforts. Expanded geographic sampling is valuable not only for detecting evolutionary lineages and identifying ESUs and inferring direct seeding but also for a wide range of other genetic analyses, including the inference of essential biodiversity variables (EBVs) related to genetic composition [64] and phylogeographic patterns.

The combination of different studies and genomic resources has allowed us to identify six ESUs (1) within the distribution range of the Carthusian pink (*D. carthusianorum* L.). The inclusion of additional samples from Austria, the Czech Republic, Italy, and Switzerland enabled the identification of a previously undetected, yet strongly divergent ESU in Italy, compared to a previous study that focused on a subset of the samples [54]. The ESUs exhibit substantial genetic differentiation (*F*_ST_: 0.17–0.43; Fig. 2) and clear phylogenetic divergence (Fig. 3), hence fulfilling the ESU criterion of strong divergence [65]. Our results indicate that the *D. carthusianorum* complex includes the six identified ESUs and two other, well-described species, *D. pontederae* and *D. giganteus* (Fig. 3). The six ESUs likely possess unique adaptive genetic diversity, as they represent deeply divergent lineages within this complex. The Eastern European species, *D. pontederae*, is adapted to low altitudes, in contrast to the partially allopatric *D. carthusianorum* ESUs, which can occur at higher elevations. Together, these results indicate that the six ESUs currently assigned to *D. carthusianorum* are most likely genetically and ecologically divergent. Given the rapid radiation rate in the genus *Dianthus*, with 1.74–16.45 species per million years [66], intrinsic or extrinsic reproductive barriers are expected to evolve quickly, which could be one explanation for the high divergence observed among the six ESUs.

Using combined genomic resources, we determined which populations were established through direct seeding in Switzerland and whether the seed sources used corresponded to the autochthonous ESUs naturally occurring at each site. We found that 48.4% of the 31 randomly selected populations in Switzerland originated from direct seeding, highlighting the substantial role of direct seeding in biodiversity promotion efforts [3, 67]. Most seeding events occurred on average ~ 11.5 years before sampling following infrastructure construction (Table 2, Additional File 1: Figure S8). This demonstrates that genomic data can detect even recent direct seeding events. Moreover, genomic data enabled the detection of seeded populations more efficiently and accurately than aerial imagery and collector assessments alone. Genomic analyses identified 48.4% of populations as seeded, compared with 35.5% by non-genomic evidence, an absolute difference of 12.9% (four additional populations), which demonstrates its enhanced sensitivity in revealing restoration activities that might otherwise go unnoticed. In contrast, one population was suggested as seeded by non-genomic evidence alone but was not supported by genomic data and was therefore excluded from the final classification of direct-seeded populations. This discrepancy could reflect restoration activities that are difficult to detect genomically, such as local hay transfer between genetically similar donor and recipient meadows, or it could result from false positives in non-genomic assessments. Overall, we observed a strong concordance of 11 populations between genomic assignments, collector information, and aerial imagery, which increases confidence in the detections and minimises the likelihood of false positives.

Our results show that substantial efforts are already being made to implement biodiversity promotion with appropriate seed material. Of the direct-seeded populations detected in Switzerland, 46.7% (7 out of 15) originated entirely from autochthonous ESUs. This is likely to aid in maintaining local genetic diversity and to mitigate risks such as maladaptation, inbreeding depression, or outbreeding depression [25–27, 68]. By contrast, 53.3% (8 of 15) of the direct-seeded populations involved at least some allochthonous input (Fig. 4b; Table 2). Of these, six were admixed populations that contained at least one allochthonous ESU, but all also included autochthonous ESUs, indicating that local material was still part of the seed source or may have introgressed from surrounding seeded or natural populations. Although we detected no introgression from allochthonous seed sources into natural populations, we did find one individual showing admixture between the highly diverged ESUs from Southern Switzerland and the Swiss Plateau (Fig. 4b). For this case, it is not possible to determine whether the admixture arose naturally, through secondary contact, or was human-mediated, for example, via seeding in a neighbouring field. In a highly genetically diverged species such as *D. carthusianorum*, hybridisation between different ESUs can lead to unpredictable outcomes. On the one hand, it may promote the masking of deleterious load and enhance fitness [69–72]. On the other hand, it may lead to maladaptation or reduced fitness due to genomic incompatibilities, resulting in outbreeding depression [25, 68]. Our analysis further revealed four distinct seed sources in Switzerland. Samples collected from populations originating from homogeneous seed sources formed star-like clusters, indicating that direct seeding has erased population structure, i.e. differentiation (Fig. 4a). The widespread use of uniform seed material can contribute to the homogenisation of regionally adapted gene pools [73] when distributed to numerous locations. Signs of this trend may already be emerging in the Swiss Plateau, where we found only one remaining natural population. While genomic data provide detailed insights into the origin and composition of seeded populations, they cannot by themselves determine whether populations are already suffering from maladaptation or outbreeding depression. However, particularly in the context of climate change, avoiding maladaptation and ensuring the use of genetically diverse, locally adapted materials is critical to support long-term population resilience and strengthen their adaptive potential [27, 74]. These findings underscore the importance of understanding evolutionary processes within species, including the formation of ESUs, to inform restoration strategies such as direct seeding, translocation, or reintroduction [75, 76].

High genetic diversity provides neutral and adaptive variation, which is fundamental for a species’ ability to respond to environmental change [28, 77]. In Switzerland, levels of genetic diversity differed significantly between four out of five ESUs (Fig. 5), which is likely due to their distinct evolutionary histories [15]. The population established with allochthonous seed material from the Eastern Europe ESU showed the lowest genetic diversity and contained among the most inbred individuals, which is most likely a consequence of genetic bottlenecks during seed multiplication [78]. However, intrinsically low natural genetic diversity cannot be excluded. In addition, we detected in populations from the Western Europe ESU a non-significant trend toward higher inbreeding in seeded compared to natural populations, which may likewise reflect the effects of repeated seed multiplication steps. These observations indicate the risk for diversity loss during seed production and emphasise the need for sustainable seed production practices, including constant monitoring and periodic refreshing of the seed sets used for propagation. The Southern Switzerland ESU exhibited relatively low genetic diversity, with a mean *F* close to zero, but included several highly inbred individuals. The overall pattern is consistent with isolation in glacial refugia followed by postglacial recolonisation, as none of these populations were seeded. However, the few individuals with high inbreeding values could be the result of small and isolated populations that have emerged due to the loss of more than 95% of dry meadows in Switzerland [45]. In the Swiss Plateau ESU, the seeded populations were outbred, with negative mean inbreeding coefficients and high genetic diversity, similar to the natural populations, suggesting that seed production did not cause inbreeding and largely preserved the genetic diversity found in natural populations.

*F* values were estimated within ESUs, but only a single population represents the Central Europe ESU, and the Eastern Europe ESU (seeded) is represented by two. In such small groups, allele frequencies are derived from fewer, more closely related individuals and fewer segregating sites, which can lead to departures from the Hardy–Weinberg equilibrium. This may bias *F* values downward, i.e. toward apparent outbreeding, making these estimates conservative relative to groups represented by more populations.

At present, we cannot infer the fitness consequences of admixture between ESUs; resolving this would require controlled crosses and common-garden experiments. From a conservation perspective, direct seeding to promote biodiversity should emphasise the consistent use of locally adapted seed provenances with high genetic diversity to enable future adaptation to a changing environment [23, 27, 79, 80]. The promotion of genetic diversity, combined with the preservation of local adaptation, can enhance both fitness and resilience in ecological restoration outcomes [79]. Providing organisms with high genetic diversity is therefore beneficial in conservation planning for promoting sustainable biodiversity [81, 82].

The integration of ddRAD and WGS data based on a SNP panel proved both feasible and highly informative. The ddRAD dataset, which included samples from outside Switzerland, extended geographic coverage, while the inclusion of WGS data provided high-quality genotypes with lower error rates, resulting in more reliable estimates of *H*_o_ (Additional File 1: Figure S3). Together, these datasets enabled us to identify allochthonous populations within Switzerland and infer their likely origin in Eastern Europe. While ddRAD sequencing remains a widely used and cost-effective method for population-level studies [34, 35], the complementary use of WGS data improves the accuracy of genetic diversity estimates.

Thanks to stringent filtering and the use of SNP panels, the overall genotyping error rates varied little between replicates of the different sequencing methods and were generally low. Regardless of the type of sequencing data, populations consistently formed the same genetic clusters. We note, however, that estimates of *H*_o_ differed significantly between ddRAD and WGS (Additional File 1: Figure S3), likely reflecting effects such as allele dropout, which are known to affect reduced-representation methods [83]. We therefore based our comparisons of *H*_o_ exclusively on populations collected in Switzerland, because of the availability of WGS data. Overall, high-quality SNP panel-based genomic analyses enable the extension of national monitoring programs for nature conservation [53, 84] across broader geographic areas, providing insights into the population structure and evolutionary history of the species across its distribution range. Such insights are essential for identifying ESUs, conservation units or management units, which form the basis for informed and effective biodiversity management [19, 75].

## Conclusions

As countries strive to meet the 2030 targets of the Global Biodiversity Framework, including the restoration of 30% of degraded ecosystems and the protection of genetic diversity [85], integrating genomic data into restoration planning is becoming essential. Our study shows that combining heterogeneous genomic resources can reliably identify the extent of direct seeding and seed provenances, detect introductions of allochthonous seed material, and resolve evolutionary lineages. An approach like this can contribute to restoration objectives by guiding the selection of regionally adapted seed sources for biodiversity promotion through revegetation, thereby preserving locally adaptive variation to enhance ecosystem resilience [86]. It also helps mitigate the risks associated with hybridisation and introgression between provenances, which may lead to maladaptation and outbreeding depression [79]. We recommend that future restoration strategies incorporate genomic data and prioritise regional seed sources, particularly for species with highly diverged evolutionary lineages, to maintain genetic diversity and adaptive potential for long-term population viability.

## Methods

### *Dianthus* samples and outgroups

**Fig. 6:**
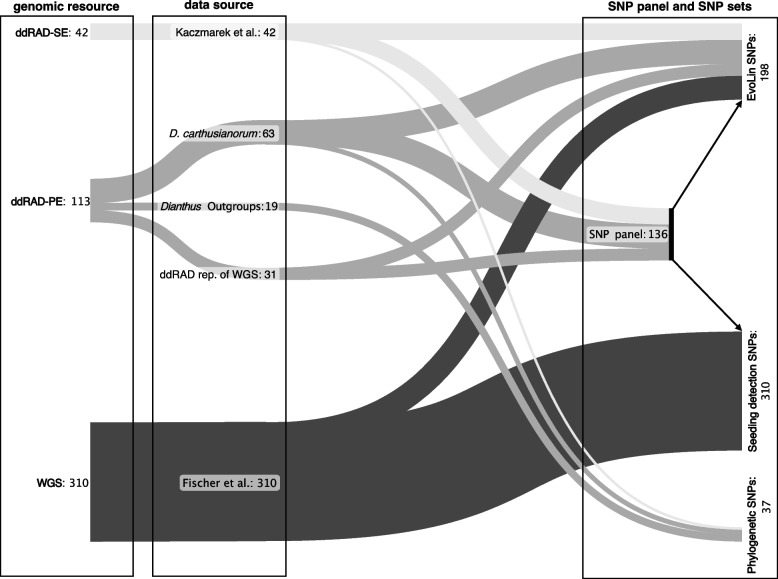
Overview of genomic resources, data sources, and their integration. Numbers indicate sample sizes. Different shades of grey represent distinct genomic resources. Arrows illustrate the SNP calling workflow: the same SNP panel was used to generate both the *EvoLin SNPs* and the *Seeding detection SNPs*. In contrast, the *Phylogenetic SNPs* were generated through de novo variant calling

To maximise geographic and genetic coverage across the species’ European distribution range, we combined data from 446 *Dianthus carthusianorum* L. individuals across 66 populations in six European countries (Austria, Czech Republic, France, Italy, Romania, and Switzerland; Fig. 1; Additional File 3). Populations were selected by incorporating available genomic data from samples with georeferenced metadata, and targeted sampling was conducted through collaborators to fill geographic gaps. To embed our samples in a broader phylogenetic context, we further included five different diploid *Dianthus* species from Europe (*D. giganteus* d'Urv*.*, *D. pontederae* A.Kern., *D. vulturius* Guss. & Ten*.*, *D. balbisii* Ser. and *D. sylvestris* Wulfen), comprising 13 individuals from nine populations (Additional File 1: Figure S6 and Additional File 3). *Dianthus sylvestris* was used as outgroup. All samples were available as sequencing data or dry leaf material and originated from different studies or projects (Fig. 6).

### De novo genome assembly of the *D. carthusianorum* reference genome

We de novo assembled a haplotype-phased reference genome from a *D. carthusianorum* individual from Grisons, Switzerland (46.84° N, 10.37° E). Sequencing and assembly were performed by Dovetail Genomics (now Cantata Bio, California, USA). Briefly, flash-frozen leaf tissue was sent to Dovetail for high molecular weight DNA extraction, library preparation for Pacific Bioscience High-fidelity (PacBio HiFi) sequencing on Sequel II, and Dovetail Omni-C library generation and paired-end sequencing. HiFi reads and Omni-C reads were used to produce haplotype-phased assemblies by using hifiasm, a haplotype-resolved de novo assembler optimised for PacBio HiFi data, with default parameters [87]. Omni-C reads were further applied for scaffolding using the Dovetail HiRise pipeline [55]. Scaffolds from the hap1 assembly with low coverage after short-read mapping were removed, resulting in 62 scaffolds used for analysis. For more detailed information, see the Supporting Methods.

### Genomic resources and sample preparation

We combined genomic resources (Fig. 6) from different sequencing techniques by producing a de novo SNP panel (see details below) tailored to integrate data from these diverse sources. The different genomic resources combined are shown in Fig. 2 and consist of double-digest restriction-site associated DNA single-end (ddRAD-SE) and paired-end (ddRAD-PE) sequencing, as well as whole-genome re-sequencing (WGS) data.

ddRAD-SE sequence data of 42 individuals were obtained as a subset of the 172 *D. carthusianorum* samples from a previous study [54] that provided broad geographic coverage across the species’ range and high-quality genomic data, making it a useful resource for assembling a representative subset of individuals. To ensure a balanced sampling design that would not bias population genetic inferences, we randomly selected three individuals from each of the 14 populations. The cited study served solely as a data source and did not guide population choice. DNA extractions for the ddRAD-SE samples in the original study were performed using the NucleoSpin Plant II, Mini kit for DNA (Macherey–Nagel, Düren, Germany).

In addition, sequence data from 113 ddRAD-PE sequenced individuals from 65 populations were obtained as part of ongoing research projects in our group to achieve a more even distribution of samples across Europe. The DNA was extracted using a customised plant extraction kit (Sbeadex™, LGC Genomics, Teddington, UK) on a KingFisher Flex instrument (Thermo Fisher Scientific, Massachusetts, USA).

For both ddRAD datasets, the DNA was quantified through QuantiFluor® ONE dsDNA dye (Promega, Madison, Wisconsin, USA) on a Spark 10 M Platereader (Tecan, Männedorf, Switzerland). Library preparation followed established ddRAD protocols [88, 89], adapted to a lower reaction volume and fewer clean-up steps: We performed double digestion on 51 ng of DNA per sample with *Eco*RI-HF (NEB R3101S) and *Taq I-v2* (NEB R0149S) restriction enzymes (0.4 µl each) from New England Biolabs (NEB, Ipswich, Massachusetts, USA) in a 22 µl reaction volume. Without a clean-up step, ligation of P1 (3 µM) and P2 (3 µM) biotin adapters (2 µl each) to the double-digested product was performed using 3 µl ATP solution (10 µM), 0.9 µl T4 ligase (NEB M0202L). The 48 individually barcoded P1 adapters allow sub-pooling of 48 samples. Double-sided size selection of these pools, aiming for ~ 550 bp library size, was done directly, without a clean-up step by adding 0.58 × AMPure XP beads (Beckman Coulter Genomics, Nyon, Switzerland), supernatant transferring and adding 0.12 × AMPure XP beads. Unique double indexes were added to sub-pools using KAPA HiFi HotStart ReadyMix (KK2602, Roche, Basel, Switzerland) via PCR (polymerase chain reaction) with 11 cycles, which was cleaned up using 0.7 × AMPure XP beads. Library size profiles were checked with an HS D1000 ScreenTape on a 4510 Tapestation (Agilent Technologies, California, USA). After multiplexing the indexed library pools, 150 bp ddRAD-SE sequencing was run on an Illumina HiSeq 3000 at the Functional Genomics Centre Zurich (FGCZ) in Switzerland; the 2 × 150 bp PE was sequenced on Illumina NovaSeq 6000 by Novogene (Novogene Company Limited in Cambridge, United Kingdom).

The 310 WGS *D. carthusianorum* samples from 31 populations originated from the Swiss pilot study for monitoring genetic diversity [53]. Populations were sampled randomly but proportional to the six biogeographic regions in Switzerland based on normalised observation data from the national database InfoFlora (https://www.infoflora.ch). The DNA of ten individuals per population was extracted and quantified as described above. The sequencing library preparation for the WGS data samples followed the NEBNext® Ultra™ II FS DNA Library Prep Kit (E7805) using a third of the specified reaction volumes. An insert size of ~ 550 bp was selected. Individuals were indexed using the NEBNext® Multiplex Oligos kit (E6440; New England Biolabs, Ipswich, USA) and equimolarly pooled. The 2 × 150 bp PE sequencing was performed on an Illumina NovaSeq 6000 by Novogene (Novogene Company Limited, Cambridge, United Kingdom), targeting 14 × sequencing coverage.

### In silico SNP panel and genotyping

Samples were demultiplexed and mapped to the *D. carthusianorum* reference genome, which was assembled in this study. The raw Illumina sequencing reads of the ddRAD datasets were demultiplexed using the Python script ‘process_radtags’ from Stacks [90]. The reads of each ddRAD and WGS sample were mapped to the reference genome using ‘BWA-mem2’ [91]. Low-quality reads were removed using ‘sambamba’ (-F "mapping_quality > = 20"; [92]).

To meet the specific analytical goals of this study, we developed three distinct SNP sets: The *EvoLin SNPs*, designed to identify evolutionary lineages and assess genetic divergence within *D. carthusianorum*; the *Phylogenetic SNPs*, intended to explore broader evolutionary relationships, including related *Dianthus* species; and the *Seeding detection SNPs*, tailored explicitly for detecting direct seeding and admixture events within Swiss populations. We computationally identified a core SNP panel from overlapping loci in ddRAD-SE and ddRAD-PE data, referred to as the SNP panel. This served as the foundation for designing three functional SNP sets. As these approaches represent only a fraction of the genome, this panel reflects the smallest genomic intersection between them. However, all these variants are also present in the WGS data. The in silico SNP panel was based on an initial variant calling of 136 individuals, including 42 ddRAD-SE individuals and 63 ddRAD-PE individuals, with three individuals per population and 31 ddRAD-PE individuals representing replicates of the WGS samples from Switzerland (Fig. 2). The BAM files of the different individuals were merged with ‘samtools’ [91]. SNPs were called using ‘freebayes’ (-m 5 -q 5 –max-complex-gap −1 –haplotype-length −1 –min-repeat-entropy 1 -V -F 0.01 –use-best-n-alleles 4; [93]). To create the SNP panel, the VCF file was filtered following the dDocent pipeline [94] that uses ‘vcftools’ [95] and ‘VCFlib’ [96]. Our filtering aimed to retain high-quality SNPs while retaining as many rare variants as possible to cover the European-wide genetic variation of *D. carthusianorum*. We filtered for a minor allele count of 1 and a minimum sequencing depth of 2, we removed individuals with more than 55% missing SNPs, we filtered for a minimum mean sequencing depth of 5, an allele balance of > 0.2 and 0.8 for heterozygous and < 0.01 respectively > 0.9 for homozygous alleles, a mapping quality between reference and alternate alleles of > 0.25 and < 1.75, a quality per depth score of > 0.2 and a maximum mean depth of 30. We kept only biallelic SNPs and excluded SNPs with more than 10% missingness.

To infer evolutionary lineages, we generated a final SNP set referred to as *EvoLin SNPs*, derived from 198 individuals (three per population across 66 populations). To ensure a balanced sampling, we used the same 136 ddRAD-SE and -PE samples as in the previously defined SNP panel and added 62 WGS samples and 31 ddRAD-WGS replicates to complete the sampling. The variant calling was performed by merging the individual.bam files of the selected samples using 'samtools' [91] and calling variants with 'freebayes' (-m 5 -q 5 –max-complex-gap −1 –haplotype-length −1 –min-repeat-entropy 1 -V -F 0.01 –use-best-n-alleles 4; [93]). Importantly, we restricted variant calling to the SNPs previously identified in the SNP panel to ensure consistency across datasets. The resulting VCF file was filtered using the same steps as the SNP panel to obtain high-quality SNPs in all samples. The resulting VCF was first processed through our standard SNP panel pipeline to retain only high-quality variants across all samples. To mitigate LD under this permissive filtering and avoid potential bias in analyses sensitive to LD, we applied a secondary LD-thinning step, retaining one SNP per 5 kb, which served as a comparison dataset to ensure consistent results.

To assess the historical evolutionary divergence of the evolutionary lineages, we conducted a phylogenetic analysis that required an additional SNP set, covering a broader taxonomic breadth, including the five closely related *Dianthus* species. We performed a de novo variant calling without using a SNP panel, based on ddRAD-SE and ddRAD-PE samples, to obtain another final SNP set called *Phylogenetic SNPs*. The *Phylogenetic SNPs* encompassed 37 individuals, two to three from each of five related *Dianthus* species and three individuals per *D. carthusianorum* ESU. To ensure accurate representation of the lineages, we only included non-admixed individuals from each ESU, as admixture could obscure phylogenetic signals. The BAM files were merged with ‘samtools’ [91], and SNPs were natively called using ‘freebayes’ (-m 5 -q 5 –max-complex-gap −1 –haplotype-length −1 –min-repeat-entropy 1 -V -F 0.01 –use-best-n-alleles 4; [93]). To finalise the SNP set, the VCF files were filtered specifically for phylogenetic analysis of more divergent taxa using ‘vcftools’ [95]. Specifically, we filtered for a minimum sequencing depth of 2, retained only biallelic SNPs, and a maximum SNP missingness of 25%. Additionally, we thinned SNPs that were closer than 5 kb to account for linked SNPs.

To identify direct seeding in Switzerland, we generated a third SNP set, called *Seeding detection SNPs*, based on the SNP panel and subsequent variant calling and filtering. The *Seeding detection SNPs* included 310 WGS samples, with ten individuals per population to detect seeded individuals, populations, and admixture levels within individuals. The variant calling and filtering of the SNP set was done in the same way as for the *EvoLin SNPs*.

### Identification of evolutionary lineages and ESUs

ESUs are generally defined by a combinatorial continuum of historical isolation (genomic divergence) and adaptive (phenotypic) divergence of lineages [19, 21, 65]. In this study, ESUs were defined primarily based on genomic evidence of historical genetic isolation and strong divergence, as phenotypic differentiation among lineages of *D. carthusianorum* can be subtle. We operationalised this genomic-based definition through integrative analyses of admixture clustering, *F*_ST_, and phylogenetic reconstruction together with closely related *Dianthus* species.

We analysed genetic structure using the *EvoLin SNPs* through ADMIXTURE [97], performing 100 independent runs for each* K* value from 1 to 10, using tenfold cross-validation and 1000 bootstraps per replicate (Additional File 1: Figure S4 and S5). Latent clustering modes were identified and summarised using the pong software [98]. To identify the optimal number of genetic clusters (*K*), we averaged the results from all 100 runs and plotted the cross-validation error and log-likelihood distribution. The optimal value for *K* was determined by inspecting cross-validation errors and log-likelihoods for different *Ks.* Additionally, we considered the ADMIXTURE pie charts, a phylogenetic tree and a pairwise fixation index (see following paragraphs) to determine the optimal *K*. Population-based ADMIXTURE pie charts were generated for visualisation using a representative run of the dominant clustering mode in R v. 4.3.2 [99] using the packages ‘mapdata’ [100], ‘marmap’ [101], and ‘rworldmap’ [102].

Phylogenetic relationships within the *EvolLin SNPs* were inferred using Randomised Axelerated Maximum Likelihood [RAxML; 103], based on a concatenated SNP alignment. We applied the GTR + GAMMA substitution model with the Lewis correction (–asc-corr = lewis; 63, [104]) to account for ascertainment bias due to the exclusion of invariable sites. Prior to analysis, we removed sites identified as invariable by RAxML. Branch support was estimated using 1000 rapid bootstrap replicates. Nodes with bootstrap support below 95% were collapsed using ‘nw_ed’ [105]. The resulting tree was rooted in FigTree [106], using populations of *Dianthus sylvestris* as outgroup, as these were inferred to show the longest cumulative branch length (see Fig. 3).

To estimate relative genetic differentiation among the different evolutionary lineages, a pairwise fixation index (*F*_ST_) was calculated using the *EvoLin SNPs* [107]. For this analysis, we included only non-admixed (i.e. < 10% admixture with any other ESU) individuals. We calculated pairwise *F*_ST_ in R using the ‘ape’ [108] and ‘SNPRelate’ packages [109].

To assess evolutionary isolation among ESUs in a broader phylogenetic context, we analysed the *Phylogenetic SNPs*, including five well-defined *Dianthus* species and three non-admixed individuals per ESU and compared tree topology and branch lengths. The phylogeny was constructed using RAxML [103] based on a concatenated SNP alignment, employing the same parameters as those described above for the *EvoLin SNPs*.

This comparative approach was essential to confirm that the evolutionary lineages and genetic clusters identified through our analyses met the criteria of significant genetic divergence and historical isolation required for classification as ESUs.

### Identification of direct seeding

To detect direct-seeded individuals and populations, we developed a sequential pipeline of different genomic approaches, which we further compared and validated with non-genomic map-based and expert-based inferences.

First, to detect allochthonous ESU seed sources, we examined the distribution of ESUs across the distribution range of *D. carthusianorum* in Europe and Switzerland using the *EvoLin SNPs*. A Swiss population was categorised as ‘seeded’ from an allochthonous ESU if it consisted of genotypes whose nearest naturally occurring, non-admixed population was located more than 100 km away, a conservative proxy for the ESU range border beyond which natural gene flow is unlikely. Populations showing ≥ 10% ancestry from another ESU in ADMIXTURE were considered admixed and were classified as containing allochthonous input if one of the contributing ESUs met this criterion.

Second, to detect direct seeding from regional homogeneous seed sources, we used the *Seeding detection SNPs* to construct an individual-based unrooted phylogenetic tree from a pairwise dissimilarity matrix calculated with the ‘SNPRelate’ [109] and ‘ape’ [108] packages in R version 4.3.2 [99]. Terminal branches were coloured by population in FigTree [106]. Populations forming star-like phylogenetic structures with minimal internal divergence, where individuals did not cluster within populations but instead grouped across populations, were classified as ‘seeded’, indicative of recent establishment from homogeneous seed sources.

Third, we also identified direct seeding by detecting artificially admixed populations using an individual-based ADMIXTURE analysis with *Seeding detection SNPs* (Additional File 1: Figure S7). Populations were classified as ‘seeded’ if they contained individuals with > 10% admixture from at least one allochthonous ESU [110]. By contrast, admixture involving only autochthonous ESUs was considered natural, reflecting contact zones between neighbouring ESUs [18]. ADMIXTURE analysis was performed in the same way as for the *EvoLin SNPs*. The number of clusters for the Swiss *Seeding Detection SNPs* was set to *K* = 5, consistent with the Europe-wide ADMIXTURE analysis of *EvoLin SNPs*. Visualisation was done in R version 4.3.2 [99].

To assess direct seeding through non-genomic tools, we used two complementary approaches. First, we analysed aerial photographs of all sampling locations to detect human-induced habitat changes, as *D. carthusianorum* is frequently seeded after infrastructure development for revegetation. We reviewed images from 1990 to 2021 using Swisstopo’s ‘Journey through time-maps’ tool (https://map.geo.admin.ch/), which provides aerial photographs at intervals of 1–5 years (longer for earlier decades). Direct seeding periods were inferred by identifying visible landscape changes, including infrastructure construction, river restoration, or other land-use modifications. The most likely motivation for biodiversity promotion measures was then assigned based on these observed changes (Additional File 1: Figure S8).

Second, during the 2021 sampling campaign, the collectors assessed whether the sampled population appeared natural or seeded. A population was categorised as direct-seeded if it consisted of deliberately introduced plants or if the seeding was intended for restoration purposes, such as following river or landscape restoration or the development of new infrastructure. To evaluate consistency across methods, we compared populations classified as ‘seeded’ genomically with those identified by aerial photographs and collector assessments. However, the final classification of seeded populations relied solely on the results of our genomic analyses.

### Genetic diversity of the natural and seeded populations in Switzerland

To assess whether there is variation in genetic diversity and inbreeding among natural, seeded and admixed populations in Switzerland, we computed observed heterozygosity (*H*_O_) and the inbreeding coefficient (*F*) using the *Seeding detection SNPs*. *H*_o_ was calculated at the individual level as the proportion of heterozygous loci per individual using ‘vcftools’ [95]. *F* was also calculated with ‘vcftools’ at the individual level within ESUs. ‘Vcftools’ computes the individual inbreeding coefficient as *F* = (*O–E*)/(*N–E*) where *O* is the number of observed homozygous sites, *E* is the number of expected homozygous sites given ESU-specific allele frequencies, and *N* is the number of sites [111]. Estimating allele frequencies within ESUs rather than across the entire dataset helped to avoid bias from the strong genetic divergence among ESUs. Downstream processing, visualisation, and statistical testing were conducted in R version 4.3.2 [99] using the packages ‘adegenet’ [112], and ‘tidyverse’ [113]. To test for differences in *H*_o_ and *F* among ESUs and between natural and seeded populations, we performed one-way ANOVAs in R after confirming normality of residuals and homogeneity of variances (Additional File 1: Figure S9a, b), followed by Tukey’s Honestly Significant Difference (HSD) post hoc tests implemented in multcompView package [114, 115].

### Evaluating error rates of combined genomic resources

The *EvoLin SNPs* (biallelic SNPs only) were used to determine if the combination of heterogeneous sequencing information was accurate. Eight technical ddRAD replicates were designed to assess the ddRAD genotyping error rate, and five technical WGS replicates were used to estimate the WGS-based genotyping error rate. In addition, 31 individuals were sequenced with both WGS and ddRAD-PE, providing 31 cross-platform technical replicates for direct comparison. All technical replicates were produced from independent DNA extractions and library preparations. The homozygous, heterozygous, and global error rates were calculated using TIGER [116]. Additionally, we compared *H*_o_ between samples genotyped with ddRAD and WGS (methods above and results in Additional File 1: Figure S3), and for consistency, all subsequent *H*_o_ estimates were based on WGS data.

## Supplementary Information

Additional file 1: Figures S1–S9, Supporting Methods, Supporting Results and Table S1. Fig. S1. GenomeScope 2.0 profile of the 21-mer frequency distribution from PacBio HiFi whole-genome reads of *Dianthus carthusianorum*. Supporting Methods: Dovetail Omni-C Library Preparation and Sequencing, Scaffolding the Assembly with HiRise, and Annotation for the *Dianthus carthusianorum *reference genome. Supporting Results: Genome assembly statistics and annotation summaries for the *Dianthus carthusianorum *reference genome. Table S1. Summary statistics of the *Dianthus carthusianorum* reference assembly (ethDiaCart_GR_1.1). Fig. S2. *K*-mer plot and link-density histogram. Fig. S3. Comparison of observed heterozygosity (*H*_O_) between ddRAD-PE and whole-genome sequencing. Fig. S4. Cross-validation scores, log-likelihoods, and admixture plots for *K* = 2–10 using *EvoLin SNPs*. Fig. S5. Population admixture patterns for *K* = 2–10 across Switzerland and Europe using *EvoLin SNPs*. Fig. S6. Sampling locations for the five *Dianthus* species. Fig. S7. Individual admixture results for *K* = 2–10 across Swiss populations using *Seeding Detection SNPs*. Fig. S8. Historical and modern aerial imagery (SWISSIMAGE Zeitreise). Fig. S9. ANOVA residual diagnostics for observed heterozygosity (*H*_O_) and inbreeding coefficient (*F*) in Swiss *Dianthus carthusianorum* individuals.

Additional file 2: Annotation of *Dianthus carthusianorum* reference genome (hap1 and hap2).

Additional file 3: Metadata of all samples.

## Data Availability

The *Dianthus carthusianorum* reference genome ethDiCart_GR_1.1 (and the corresponding raw reads) can be found at NCBI (www.ncbi.nlm.nih.gov) under PRJNA1259412 [117]. The second haplotype assembly (hap2) is deposited under accession PRJNA1262190 [118]; this assembly was generated alongside hap1 but was not included in the analyses presented here. ddRAD raw reads of *D. carthusianorum*, and *D. vulturius* are available at NCBI under PRJNA1264743 [119]. On ENA (www.ebi.ac.uk), the ddRAD raw reads of (DIA_GIGA_EL_EVROS_001: ERS18350668; DIA_GIGA_RO_CHEIL_001: ERS18350666; DIA_GIGA_RO_CHEIL_002: ERS18350667), *D. pontederae* (DIA_PON_DE_BAYRE_001: ERS18350692; DIA_PON_DE_BAYRE_002: ERS18350693), and (DIA_SYL_CH_TSANF_001: ERS7647682; DIA_SYL_CH_VAREN_003: ERS7647743) are listed [120]. VCF files (SNP sets and SNP panel) and the reference genome used for mapping (CARY_DIA_CAR_CH_CHANT_001_HiFi_OMNI-C_HAP1_COR.fasta), are available at the Dryad digital repository under 10.5061/dryad.c866t1gk1 [121] and only differ in scaffold naming and orientation from ethDiCart_GR_1.1.

## Abbreviations

ANOVA: Analysis of Variance
BUSCO: Benchmarking Universal Single-Copy Orthologs
CBD: Convention on Biological Diversity
COP15: 15th Conference of the Parties
ddRAD: Double-digest Restriction-site Associated DNA
ddRAD-PE: Double-digest Restriction-site Associated DNA, paired-end protocol
ddRAD-SE: Double-digest Restriction-site Associated DNA, single-end protocol
EBV: Essential Biodiversity Variable
ESU: Evolutionarily Significant Unit
FGCZ: Functional Genomics Centre Zurich
*F*: Individual-level inbreeding coefficient
*F*_ST_: Fixation index
GBF: Global Biodiversity Framework
HiFi: High-fidelity (PacBio sequencing)
*H*_O_: Observed heterozygosity
HTS: High-Throughput Sequencing
INSCD: International Nucleotide Sequence Database Collaboration
LD: Linkage Disequilibrium
PCR: Polymerase Chain Reaction
RAD-Seq: Restriction-site Associated DNA sequencing
RAxML: Randomised Axelerated Maximum Likelihood
SDG: Sustainable Development Goal
SNP: Single Nucleotide Polymorphism
WGS: Whole-Genome re-Sequencing
