# Supplementary material for: Genomic identification of direct seeding and evolutionary lineages by combining heterogeneous genomic resources
Source: BMC Biol. 2025 Dec 12;24:14. doi: 10.1186/s12915-025-02476-5 (PMC12817430; doi:10.1186/s12915-025-02476-5)
Supplement: Supplementary file 1 — Additional file 1: Figures S1–S9, Supporting Methods, Supporting Results and Table S1. Fig. S1. GenomeScope 2.0 profile of the 21-mer frequency distribution from PacBio HiFi whole-genome reads of Dianthus carthusianorum. Supporting Methods: Dovetail Omni-C Library Preparation and Sequencing, Scaffolding the Assembly with HiRise, and Annotation for the Dianthus carthusianorum reference genome. Supporting Results: Genome assembly statistics and annotation summaries for the Dianthus carthusianorum reference genome. Table S1. Summary statistics of the Dianthus carthusianorum reference assembly (ethDiaCart_GR_1.1). Fig. S2. K-mer plot and link-density histogram. Fig. S3. Comparison of observed heterozygosity (HO) between ddRAD-PE and whole-genome sequencing. Fig. S4. Cross-validation scores, log-likelihoods, and admixture plots for K = 2–10 using EvoLin SNPs. Fig. S5. Population admixture patterns for K = 2–10 across Switzerland and Europe using EvoLin SNPs. Fig. S6. Sampling locations for the five Dianthus species. Fig. S7. Individual admixture results for K = 2–10 across Swiss populations using Seeding Detection SNPs. Fig. S8. Historical and modern aerial imagery (SWISSIMAGE Zeitreise). Fig. S9. ANOVA residual diagnostics for observed heterozygosity (HO) and inbreeding coefficient (F) in Swiss Dianthus carthusianorum individuals. [file 12915_2025_2476_MOESM1_ESM.docx]

**Additional File 1 for:**

**Genomic identification of direct seeding and evolutionary lineages by combining heterogeneous genomic resources**

Jasmine Noëlle Tschan, Oliver Reutimann, Simone Fior, Amandine Cornille, Anamaria Roman, Tudor-Mihai Ursu, Alex Widmer & Martin C. Fischer

**Table of Contents**

[FIGURE S1: GenomeScope 2.0 profile of the 21‑mer frequency distribution from PacBio HiFi whole‑genome reads of *Dianthus carthusianorum*	3](#_Toc214347976)

[SUPPORTING METHODS: Dovetail Omni-C Library Preparation and Sequencing, Scaffolding the Assembly with HiRise, and Annotation for the *Dianthus carthusianorum* reference genome.	3](#_Toc214347977)

[SUPPORTING RESULTS: Genome assembly statistics and annotation summaries for the *Dianthus carthusianorum* reference genome	4](#_Toc214347979)

[TABLE S1: Summary statistics of the reference assembly of *Dianthus carthusianorum* ethDiaCart_GR_1.1.	5](#_Toc214347981)

[FIGURE S2: *K*-mer plot and link density histogram	6](#_Toc214347982)

[FIGURE S3: Difference in observed heterozygosity (*H*_O_) between ddRAD-PE and WGS.	6](#_Toc214347983)

[FIGURE S4: Cross-validation, Log-likelihood and admixture analysis from *K* = 2 – 10 from the *EvoLin SNPs*.	8](#_Toc214347984)

[FIGURE S5: Population admixture for K = 2 – 10 across Switzerland and Europe using the *EvoLin SNPs*	11](#_Toc214347985)

[FIGURE S6: Sample origin of the five *Dianthus* species	12](#_Toc214347986)

[FIGURE S7: Individual admixture results from *K* = 2 – 10 across Swiss populations using the *Seeding Detection SNPs*	13](#_Toc214347987)

[FIGURE S8: Aerial imagery (SWISSIMAGE Zeitreise; https://map.geo.admin.ch/)	45](#_Toc214347988)

[FIGURE S9: ANOVA residual diagnostics of observed heterozygosity (*H*_O_) and inbreeding (*F*) in Swiss *Dianthus carthusianorum* individuals	46](#_Toc214347989)

# **FIGURE S1:** GenomeScope 2.0 profile of the 21‑mer frequency distribution from PacBio HiFi whole‑genome reads of Dianthus carthusianorum. The GenomeScope illustrates the empirical histogram and model fit used to infer genome size, repeat content, and heterozygosity.

# **SUPPORTING METHODS**

# *Dianthus carthusianorum* reference genome

**Dovetail Omni-C Library Preparation and Sequencing**

For each Dovetail Omni-C library, performed by Dovetail Genomics (now Cantata Bio, California, USA), chromatin was fixed in place with formaldehyde in the nucleus and then extracted. Fixed chromatin was digested with DNAse I, chromatin ends were repaired and ligated to a biotinylated bridge adapter, followed by proximity ligation of the adapter-containing ends. After proximity ligation, crosslinks were reversed, and the DNA was purified. Purified DNA was treated to remove biotin that was not internal to the ligated fragments. Sequencing libraries were generated using NEBNext Ultra enzymes and Illumina-compatible adapters. Biotin-containing fragments were isolated using streptavidin beads before PCR enrichment of each library. The library was sequenced on an Illumina HiSeq X platform to achieve approximately 30x sequence coverage. Subsequently, HiRise utilised MQ>50 reads for scaffolding.

**Scaffolding the Assembly with HiRise**

Dovetail Genomics (now Cantata Bio, California, USA) first generated a *de novo* genome assembly using hifiasm with long-read sequencing data from Pacific Biosciences (PacBio) high-fidelity (HiFi) reads. This draft assembly, together with proximity ligation data from Dovetail OmniC library reads were used as input data for HiRise, a software pipeline explicitly designed for using proximity ligation data to scaffold genome assemblies. The OmniC library sequences, which capture physical proximity between DNA fragments in the nucleus, were aligned to the draft input assembly using bwa (Burrows-Wheeler Aligner; <https://github.com/lh3/bwa>). The separations of Dovetail OmniC read pairs mapped within draft scaffolds were analysed by HiRise to produce a likelihood model for genomic distance between read pairs, and the model was used to identify and break putative misjoins, to score prospective joins, and make joins above a threshold.

The reference genome used for mapping the *Dianthus* reads in this paper is on Dryad (<https://doi.org/10.5061/dryad.c866t1gk1>) and corresponds to haplotype 1 (hap1). It differs only in scaffold naming and orientation (CARY_DIA_CAR_CH_CHANT_001_HiFi_OMNI-C_HAP1_COR.fasta) compared to the assembly uploaded on NCBI (ethDiaCart_GR_1.1; PRJNA1259412). The assembler produced a haplotype-resolved draft with two phased assemblies (‘hap1’ and ‘hap2’), each representing one parental haplotype; hap2 was retained but not used for downstream analyses.

**Annotation**

We identified repeat families in the genome assemblies of *Dianthus carthusianorum* *de novo* classified using the RepeatModeler software package version 2.0.1. RepeatModeler depends on the programs RECON version 1.08 and RepeatScout version 1.0.6 for the *de novo* identification of repeats within the genome. The custom repeat library obtained from RepeatModeler was used to discover, identify, and mask repeats in the assembly file using RepeatMasker version 4.1.0 (<http://repeatmasker.org>). Coding sequences from *Arabidopsis thaliana*, *Beta vulgaris*, *Chenopodium quinoa* and *Heliosperma pusillum* were used to train the initial ab initio model for *Dianthus carthusianorum* using the AUGUSTUS software with version 2.5.5. Six rounds of prediction optimisation were done with the software package provided by AUGUSTUS. The identical coding sequences were also used to train a separate ab initio model for *Dianthus carthusianorum* using SNAP version 2006-07-28. RNA-Seq reads from six independent RNA-Seq rounds of leaf and flower tissue were mapped onto the genome using the STAR aligner software with version 2.7 and intron hints generated with the bam2hints tools within the AUGUSTUS software. MAKER, SNAP, and AUGUSTUS, with intron-exon boundary hints provided by RNA-Seq, were then used to predict genes in the repeat-masked reference genome. To guide the prediction process, Swiss-Prot peptide sequences from the UniProt database (<https://www.uniprot.org/>) were downloaded and used in conjunction with protein sequences from *Arabidopsis thaliana*, *Beta vulgaris*, *Chenopodium quinoa,* and *Heliosperma pusillum* to generate peptide evidence in the Maker pipeline. Only genes predicted by both SNAP and AUGUSTUS software were retained in the final gene sets. To help assess the quality of the gene prediction, AED scores were generated for each predicted gene as part of the MAKER pipeline. Genes were further characterised for their putative function by performing a BLAST search of the peptide sequences against the UniProt database. tRNA was predicted using the software tRNAscan-SE version 2.05.

# **SUPPORTING RESULTS**

# *Dianthus carthusianorum* reference genomes

Haplotype 2 (ethDiaCart_GR_1.2) can be found on NCBI and is 502 Mb, consisting of 268 scaffolds, 15 of which are anchored on chromosomes (NCBI, PRJNA1262190).

The functional annotation of both haplotypes can be found in Additional File 2.

# **TABLE S1:** Summary statistics of the reference assembly of *Dianthus carthusianorum* ethDiaCart_GR_1.1.

|  | **hap1 (ethDiaCart_GR_1.1)** |
| --- | --- |
| **Size (bp)** | 498,671,606 |
| **Scaffolds** | 62 |
| **Ns** | 6,900 |
| **Gaps** | 69 |
| **Scaffold N50** | 33,218,876 |
| **Scaffold N90** | 30,263,282 |
| **Scaffold L90** | 14 |
| **Largest scaffold** | 39,410,985 |
| **Assembly BUSCO (eudicots_odb10)** | C:93.5% [S:88.2%, D:5.3%], F:1.3%, M:5.2%, n:2326 |
| **Contig N50** | 11,463,605 |
| **Contig N90** | 4,014,208 |
| **Contig L90** | 42 |
| **Largest contig** | 25,413,205 |
| **QV** | 60.88 |
| ***k*-mer completeness** | 75.37 |
| **Average GC content** | 37% |
| **Total annotated genes** | 40,047 |
| **Mean Total CDS Length** | 1,755 |
| **Annotation BUSCO (eudicots_odb10)** | C:89.6% [S:86.3%, D:3.3%], F:1.0%, M:9.4%, n:2326 |

# **FIGURE S2:** *K*-mer plot and link density histogram. a) *K*-mer plot for the primary haplotype assembly ethDiaCart_GR_1.1 hap1. a) The assembly *k*-mer copy number spectrum shows low level of duplications within the assembly. Read-only *k*-mers in the single-copy peaks are from alternate haplotypes. b) Link density histogram for ethDiaCart_GR_1.1 hap 1.

**FIGURE S3:** Difference in observed heterozygosity (*H*_O_) between ddRAD-PE and WGS. The significant difference in *H*_O_ means (*p* = 8.7e^-05^, Wilcoxon rank sum test) between the sequencing types is 3.22%.

# **FIGURE S4:** Cross-validation, Log-likelihood and admixture analysis from *K* = 2 – 10 from the *EvoLin SNPs*. (a) Cross-validation error (top) and log-likelihood values (bottom) for the original *EvoLin SNPs* containing 42,466 SNPs. (b) Corresponding cross-validation and log-likelihood plots for the LD-thinned *EvoLin SNPs* with 6,307 SNPs. (c) Individual admixture proportions inferred from the original *EvoLin SNPs*; the dotted line indicates the best fitting *K = 6*. (d) Individual admixture proportions inferred from the LD-thinned *EvoLin SNPs*; the dotted line indicates the chosen *K*. (e) Scatterplot comparing individual ancestry proportions (Q-values) between the original and LD-thinned *EvoLin SNPs*, illustrating consistency between the two analyses.

# **FIGURE S5:** Population admixture for K = 2 – 10 across Switzerland and Europe using the *EvoLin SNPs*. The number of runs determines the support for the given grouping.: *K* = 2: 100/100; *K* = 3: 77/100; *K* = 4: 61/100; *K* = 5: 85/100; *K* = 6: 86/100; *K* = 7: 61/100; *K* = 8: 54/100; *K* = 9: 24/100; *K* = 10: 19/100.

# **FIGURE S6:** Sample origin of the five *Dianthus* species. Colours indicate species, and labels refer to individual sample names. The naming convention is as follows: DIA = *Dianthus*, followed by a species abbreviation, a country code, a population identifier, and an individual number (e.g., DIA_SYL_CH_TSANF_001 = *D. sylvestris*, Switzerland, population from Tsanfleuron, individual 001). In total, 13 individuals from 9 populations are shown. Additional information and coordinates are provided in Table S1.

**FIGURE S7:** Individual admixture results from *K* = 2 – 10 across Swiss populations using the *Seeding Detection SNPs*. (a) Individual-level ancestry estimates based on the full *Seeding Detection SNPs* (33,725 SNPs); the dotted line indicates the chosen *K*. (b) Corresponding admixture proportions obtained from the LD-thinned SNP set (6,106 SNPs); the dotted line indicates the best fitting *K* = 5. (c) Scatterplot comparing Q-values between the original and LD-thinned SNP sets.

# **FIGURE S8:** Aerial imagery (SWISSIMAGE Zeitreise; <https://map.geo.admin.ch/>). The aerial photographs are surrounded by green for natural populations and red for direct seeding populations. (a) Population 01 (CH_DOMLE), (b) Population 02 (CH_CARTI), (c) Population 03 (CH_BIERE), (d) Population 04 (CH_PREMI), (e) Population 05 (CH_DITTI), (f) Population 06 (CH_LOMMI), (g) Population 07 (CH_COURT), (h) Population 08 (CH_MOESC), (i) Population 09 (FRAUE), (j) Population 10 (CH_VILTE), (k) Population 11 (CH_GRIMM), (l) Population 12 (CH_PFUNG), (m) Population 13 (CH_GLSUE), (n) Population 14 (CH_VOLKE), (o) Population 15 (CH_REMIG), (p) Population 16 (CH_ORVIN), (q) Population 17 (CH_VECHI), (r) Population 18 (CH_VALMU), (s) Population 19 (CH_BAERE), (t) Population 20 (CH_BETTS), (u) Population 21 (CH_MONOB), (v) Population 22 (CH_BAGNE), (w) Population 23 (CH_GRAEC), (x) Population 24 (CH_VISPE), (y) Population 25 (CH_GRENG), (z) Population 26 (CH_WASSE), (aa) Population 27 (CH_HOSPE), (bb) Population 28 (CH_BIASC), (cc) Population 29 (CH_MAGGI), (dd) Population 30 (CH_DALPE), (ee) Population 31 (CH_BUSEN).

# **FIGURE S9:** ANOVA residual diagnostics of **observed heterozygosity (*H*_O_)** and inbreeding (*F*) in Swiss *Dianthus carthusianorum* individuals**.** (a) Model of individual observed heterozygosity (*H*_O_) and (b) inbreeding coefficient (*F*) based on *Seeding Detection SNPs*. Each model assesses variation across evolutionary significant units (ESUs) and between natural and seeded populations.
